# Supplementary figures and images for: Cell segmentation and tracking using CNN-based distance predictions and a graph-based matching strategy
Source: PLoS One. 2020 Dec 8;15(12):e0243219. doi: 10.1371/journal.pone.0243219 (PMC7723299; doi:10.1371/journal.pone.0243219)

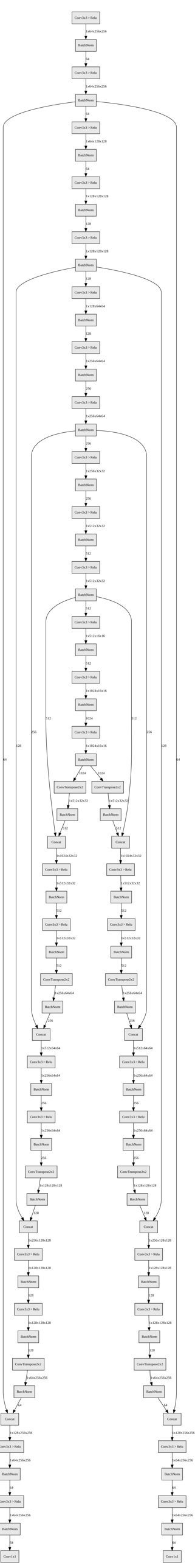

Supplement: S1 Fig — (PDF) [file pone.0243219.s001.pdf]

## CTC Training Set

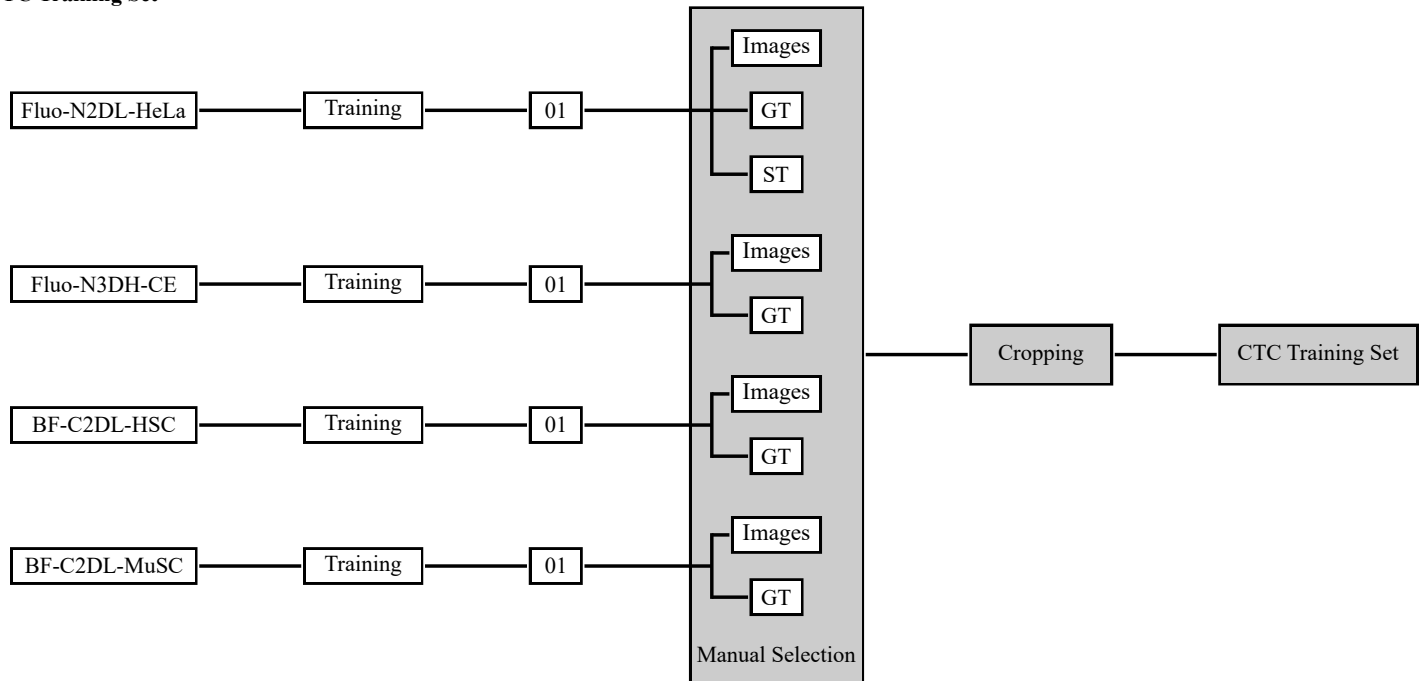

## Fluo-N2DL-HeLa Test Set

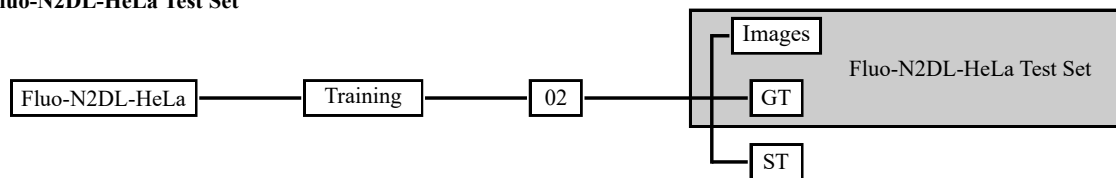

## Fluo-N3DH-CE Test Set

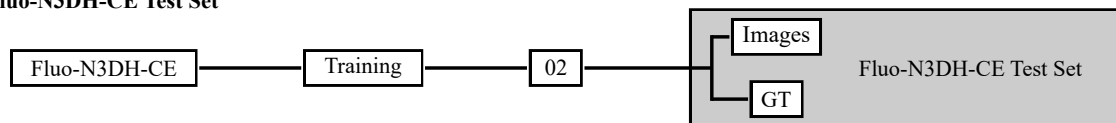

## BF-C2DL-HSC Test Set

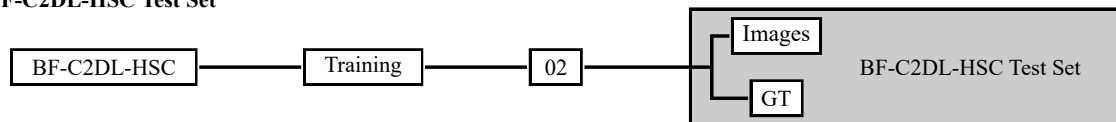

## BF-C2DL-MuSC Test Set

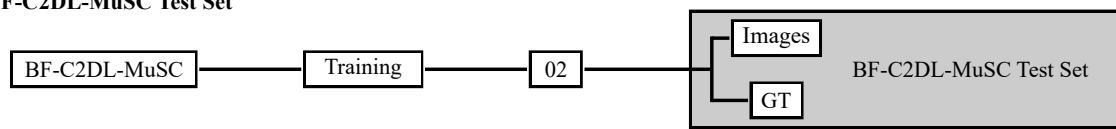

Supplement: S2 Fig — The ground truths of the challenge sets from the Cell Tracking Challenge are not publicly available. Thus, the two training data sets are split into a training set and cell type specific test sets for our segmentation experiments. For the training data set only fully annotated segmentation GTs and good quality STs can be used to train models well. For evaluation, all segmentation and detection GTs can be used. (PDF) [file pone.0243219.s002.pdf]

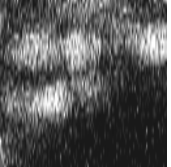

(a) Test Image

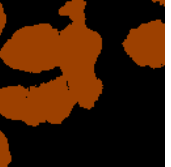

(b) Boundary

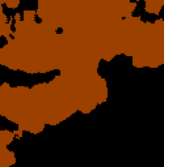

(c) Border

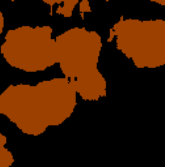

(d) Adapted Border

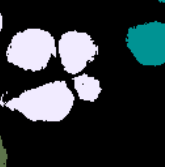

(e) Dual U-Net

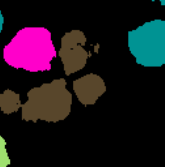

(f) J4

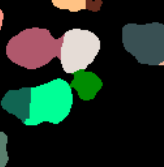

(g) Proposed

Supplement: S3 Fig — Only the proposed method is able to prevent from merging close cells in late frames after many cell divisions. Note: this is a low-resolution 3D data set and the erroneous merging of cells can result from any of the slices a cell appears. (PDF) [file pone.0243219.s003.pdf]
