# Supplementary material for: Cell segmentation and tracking using CNN-based distance predictions and a graph-based matching strategy
Source: PLoS One. 2020 Dec 8;15(12):e0243219. doi: 10.1371/journal.pone.0243219 (PMC7723299; doi:10.1371/journal.pone.0243219)
Supplement: S1 File — (PDF) [file pone.0243219.s004.pdf]

Cell segmentation and tracking using CNN-based distance predictions and a graph-based matching strategy

Supplementary file S1: post-processing and loss functions of the compared methods

Tim Scherr[1]*, Katharina Löffler [1,2], Moritz Böhland[1], Ralf Mikut[1]

1 Institute for Automation and Applied Informatics, Karlsruhe Institute of Technology, Eggenstein-Leopoldshafen, Germany, **2** Institute of Biological and Chemical Systems – Biological Information Processing, Karlsruhe Institute of Technology, Eggenstein-Leopoldshafen, Germany

* tim.scherr@kit.edu

This supplementary file describes the post-processing of the compared methods in detail. The compared methods and our proposed method are based on a seed and mask extraction which are used for a watershed segmentation.

Boundary method

The boundary method (BM) predicts a three channel output (background, cell, boundary). Activation function is the softmax activation and a weighted sum of Dice loss (channel-wise) and cross entropy loss is used to train this method. For the watershed segmentation binary masks $\mathbf{P}_{\text{BM,mask}}$ and seeds $\mathbf{P}_{\text{BM,seeds}}$ are extracted from the single channels $\mathbf{P}_{\text{BM}}(x)$ with $x \in \{\text{background, cell, boundary}\}$:

$$p^{ij}_{\text{BM,mask}} = \begin{cases} 1 & \text{if } \arg\max_x p^{ij}_{\text{BM}}(x) = \text{cell}\,, \\ 0 & \text{else}\,, \end{cases} \tag{s1}$$

$$\mathbf{P}_{\text{BM,seeds}} = \mathbf{P}_{\text{BM}}(\text{cell}) \cdot \left[1 - \mathbf{P}_{\text{BM}}(\text{boundary})\right] > 0.5\,. \tag{s2}$$

$p^{ij}_{\text{BM,mask}}$ represents the value of $\mathbf{P}_{\text{BM,mask}}$ at pixel (i, j). The resulting mask is equal to one at pixels with maximum prediction in the cell channel. For 3D data, Eq. s1 needs to be adapted to the voxel coordinates.

Border method

The border method (BoM) predicts a three channel output (background, cell, border). Activation function is the softmax activation and a weighted sum of Dice loss (channel-wise) and cross entropy loss is used to train this method. For the watershed segmentation binary masks $\mathbf{P}_{\text{BoM,mask}}$ and seeds $\mathbf{P}_{\text{BoM,seeds}}$ are extracted from the single channels $\mathbf{P}_{\text{BoM}}(x)$ with $x \in \{\text{background, cell, border}\}$:

$$p^{ij}_{\text{BoM,mask}} = \begin{cases} 1 & \text{if } \arg\max_x p^{ij}_{\text{BM}}(x) = \text{cell}\,, \\ 0 & \text{else}\,, \end{cases} \tag{s3}$$

$$\mathbf{P}_{\text{BoM,seeds}} = \mathbf{P}_{\text{BoM}}(\text{cell}) \cdot \left[1 - \mathbf{P}_{\text{BoM}}(\text{border})\right] > 0.5\,. \tag{s4}$$

$p^{ij}_{\text{BoM,mask}}$ represents the value of $\mathbf{P}_{\text{BoM,mask}}$ at pixel (i, j). The resulting mask is equal to one at pixels with maximum prediction in the cell channel. For 3D data, Eq. s3 needs to be adapted to the voxel coordinates.

Adapted border method

The adapted border method (ABoM) predicts a single channel output (cell) used for the mask creation and a three channel output (background, eroded cell, adapted border) used for seed detection. The sigmoid activation function is applied to the single channel cell output and the softmax activation to the three channel output. A weighted sum of Dice loss and cross entropy loss is used to train both outputs. The total loss function is the sum of the loss functions of each output. For the watershed segmentation binary masks $\mathbf{P}_{\text{ABoM,mask}}$ and seeds $\mathbf{P}_{\text{ABoM,seeds}}$ are extracted from the single channels $\mathbf{P}_{\text{ABoM}}(x)$ with $x \in \{\text{cell, background, eroded cell, adapted border}\}$:

$$\mathbf{P}_{\text{ABoM,mask}} = \mathbf{P}_{\text{ABoM}}(\text{cell}) > 0.5, \tag{s5}$$

$$\mathbf{P}_{\text{ABoM,seeds}} = \mathbf{P}_{\text{ABoM}}(\text{eroded cell}) \cdot \left[1 - \mathbf{P}_{\text{ABoM}}(\text{adapted border})\right] > 0.5. \tag{s6}$$

Dual U-Net method

The Dual-U-Net method predicts a single channel cell distance output (linear activation), a single channel boundary output (sigmoid activation) and a single channel segmentation map output (sigmoid activation). To learn the cell distances a mean square error loss is used, and for each other output a weighted sum of Dice loss and cross entropy loss. The total loss function is a weighted sum of the three losses. The weighting is needed to avoid that only one loss is minimized and was adjusted to the CTC training set.

The intermediate cell distance and boundary outputs are only needed in the training process. For inference, only the segmentation output based on the intermediate predictions is used. In the corresponding publication, no specific post-processing is mentioned. However, we applied a simple morphological erosion after thresholding the segmentation map which reduces the merging of cells in some cases. To get the initial cell shapes we applied a watershed segmentation to the thresholded segmentation map with the eroded cells as seeds.

J4 method

The J4 method predicts a four channel output (background, cell, touching, gap). As activation function softmax activation is used. To train the network, a weighted sum of cross entropy loss and $\boldsymbol{J}$ regularization loss is used as loss function. Instead of using the cross entropy loss definition of the corresponding publication, we used a PyTorch implementation which is numerically more stable. Since the $\boldsymbol{J}$ regularization loss was much larger than the cross entropy loss on our data (regardless of the cross entropy implementation) we scaled it in a way that the $\boldsymbol{J}$ regularizaton loss was the dominating loss part after minimizing the cross entropy loss, as suggested in the corresponding publication.

For the watershed segmentation binary masks $\mathbf{P}_{\text{J4,mask}}$ and seeds $\mathbf{P}_{\text{J4,seeds}}$ are extracted from the single channels $\mathbf{P}_{\text{J4}}(x)$ with $x \in \{\text{background, cell, touching, gap}\}$

$$p^{ij}_{\text{J4,mask}} = \begin{cases} 1 & \text{if } \arg\max_x p^{ij}_{\text{J4}}(x) = \text{cell}\,, \\ 1 & \text{if } \arg\max_x p^{ij}_{\text{J4}}(x) = \text{touching}\,, \\ 0 & \text{else}\,, \end{cases} \tag{s7}$$

$$\mathbf{P}_{\text{J4,seeds}} = \mathbf{P}_{\text{J4}}(\text{cell}) \cdot \left[1 - \mathbf{P}_{\text{J4}}(\text{touching})\right] \cdot \left[1 - \mathbf{P}_{\text{J4}}(\text{gap})\right] > 0.5\,. \tag{s8}$$

$p^{ij}_{\text{J4,mask}}$ represents the value of $\mathbf{P}_{\text{J4,mask}}$ at pixel (i, j). The resulting mask is equal to one at pixels with maximum prediction in the cell channel or in the touching channel (touching class can only occur within cells, gaps within background). For 3D data, Eq. s7 needs to be adapted to the voxel coordinates.

We also tested to just use the arg max of the cell class but this resulted in some additional merged cells. Since merged cells are penalized stronger by the metric than false positives/splits the method benefits from our post-processing in our comparison.
