# Supplementary material for: Cell segmentation and tracking using CNN-based distance predictions and a graph-based matching strategy
Source: PLoS One. 2020 Dec 8;15(12):e0243219. doi: 10.1371/journal.pone.0243219 (PMC7723299; doi:10.1371/journal.pone.0243219)
Supplement: S1 Table — Especially for the HeLa cells less erroneously merged cells occur compared to using only the predicted cell distance information. This enables the proposed method to be a good generalist in our comparison. (PDF) [file pone.0243219.s005.pdf]

	**BF-C2DL-HSC**	**BF-C2DL-MuSC**	**Fluo-N2DL-HeLa**	**Fluo-N3DH-CE**
Resolved merges	$< 1\,\%$	$14\,\%$	$19\,\%$	$2\,\%$
